# Response of the Indian summer monsoon to global warming, solar geoengineering and its termination

**DOI:** 10.1038/s41598-021-89249-6

**Published:** 2021-05-07

**Authors:** Mansi Bhowmick, Saroj Kanta Mishra, Ben Kravitz, Sandeep Sahany, Popat Salunke

**Affiliations:** 1 Centre for Atmospheric Sciences, Indian Institute of Technology Delhi (IIT Delhi), New Delhi, 110016 India; 2 Department of Earth and Atmospheric Sciences, Indiana University, Bloomington, IN USA; 3 Atmospheric Sciences and Global Change Division, Pacific Northwest National Laboratory, Richland, WA USA

**Keywords:** Climate sciences, Applied physics

## Abstract

The response of the Indian Summer Monsoon (ISM) to global warming, solar geoengineering and its termination is examined using the multi-model mean of seven global climate model simulations from G2 experiment of the Geoengineering Model Intercomparison Project. Under the global warming scenario, land–ocean temperature contrasts and low-level monsoon circulation progressively strengthen accompanied by enhanced precipitation over the Indian subcontinent. Notably, in the solar geoengineered scenario, marginal surface cooling is projected over the majority of the ISM region, and there is strengthening of both upper and lower level circulation. However, preferential precipitation near Western Ghats leads to dry bias over majority of Indian land. Upon the termination of the geoengineering, the climatic conditions—temperature, precipitation, winds and moisture would abruptly change to what it would have been under the global warming scenario. Thus, this may be important to note that such changes may need attention for the future mitigation and adaptation purposes if solar geoengineering is required to implement in future.

## Introduction

Global warming is causing widespread effects throughout the globe^1–3^. However, global efforts to reduce greenhouse gas emissions have not yet been successful to provide confidence that dangerous climate change can be avoided. Additional efforts may be required to quickly offset the global warming. Alternative approaches to control the climate change, several schemes have been proposed^4–6^. The idea of controlling solar radiation through sulphate aerosols is due to the observation of Mt. Pinatabu eruption^7^ that was potentially effective in reducing global warming. But, a potential risk is how the climate system would respond if geoengineering were suddenly terminated^8–11^. Although the results of geoengineering (reduction in global warming^7^) is known but process of its implementation is still uncertain. There could be many different kinds^12, 13^ of bad climatic implications during its implementation.

Solar radiation management via stratospheric sulfate aerosols is a geoengineering method^5, 6^ and if a large amount of solar geoengineering is applied, the termination effect that is swift rise in temperature to a much warmer climate seems to be inevitable^13^.

In the recent past, climate modeling groups joined a coordinated effort, now called the Geoengineering Model Intercomparison Project (GeoMIP), to conduct the same idealized geoengineering simulations, enabling scientists to obtain more robust conclusions about potential effects of the geoengineering^14, 15^. In the present study, we utilized the model outputs from GeoMIP experiment G2, in which, starting from a preindustrial baseline (piControl), the CO_2_ concentration was increased by 1% per year (the corresponding simulation with only CO_2_ increase and no geoengineering is referred to as 1pctCO2), while total solar irradiance was reduced commensurately to offset global mean temperature change. This was conducted for 50 years, and then geoengineering was abruptly terminated, and the model was run for an additional 20 years. This enables modeling groups to capture the changing climate effects due to gradual ramp-up of geoengineering, as well as the termination effect. Table 1 lists seven Earth System models that participated in all three simulations (piControl, 1pctCO2, and G2), as well as the variables from each model that are analyzed in this study.

**Table1:** List of the Earth System Models used in this study and their details.

S. no	Model name	Resolution (lon × lat) in degrees	Experiments simulated	Parameters analysed
1	BNU-ESM	2.8 × 2.8	piControl 1pctCO2 G2	Temperature, precipitation, wind, specific humidity
2	CCSM4	1.25 × 0.94	piControl 1pctCO2 G2	Temperature, precipitation, wind, specific humidity
3	CESM-CAM5.1-FV	1.25 × 0.9	piControl 1pctCO2 G2	Temperature, precipitation, wind, specific humidity
4	HadGEM2-ES	1.875 × 1.25	piControl 1pctCO2 G2	Temperature, precipitation, wind, specific humidity
5	IPSL-CM5A-LR	3.75 × 1.8	piControl 1pctCO2 G2	Temperature, precipitation, wind, specific humidity
6	MIROC-ESM	2.8 × 2.8	piControl 1pctCO2 G2	Temperature, precipitation, wind, specific humidity
7	MPI-ESM-LR	1.9 × 1.9	piControl 1pctCO2 G2	Temperature, precipitation, wind, specific humidity

Using data from multi-model simulations for solar geoengineering (G2), global warming (1pctCO2), and pre-industrial control (piControl) simulations, this paper provides a detailed description of the changes in temperature, wind speed, moisture, and precipitation for different parts of the Indian Summer Monsoon (ISM) region (15S–45 N, 40E–120E) in response to idealized CO2 warming along with deployment and termination of solar geoengineering. The region affected by the ISM is the habitat for more than 1 billion people, and the summer monsoon brings in nearly 80% of annual rainfall to this region^16^. Any changes to the monsoon, through climate change^16^ or geoengineering^17, 18^, would profoundly impact economic and food security^19^ of this region. In past, the effect of solar geoengineering on ISM has been briefly discussed in few studies^17, 18^ and the termination effect of solar geoengineering is also not new. However, considering the large population affected by ISM, this study has regional implications, not only to the end product of the application of solar geoengineering but also to the intermediate climatic conditions and processes occurring during the implementation of geoengineering.

## Changes in climatological features

**Figure 1:**
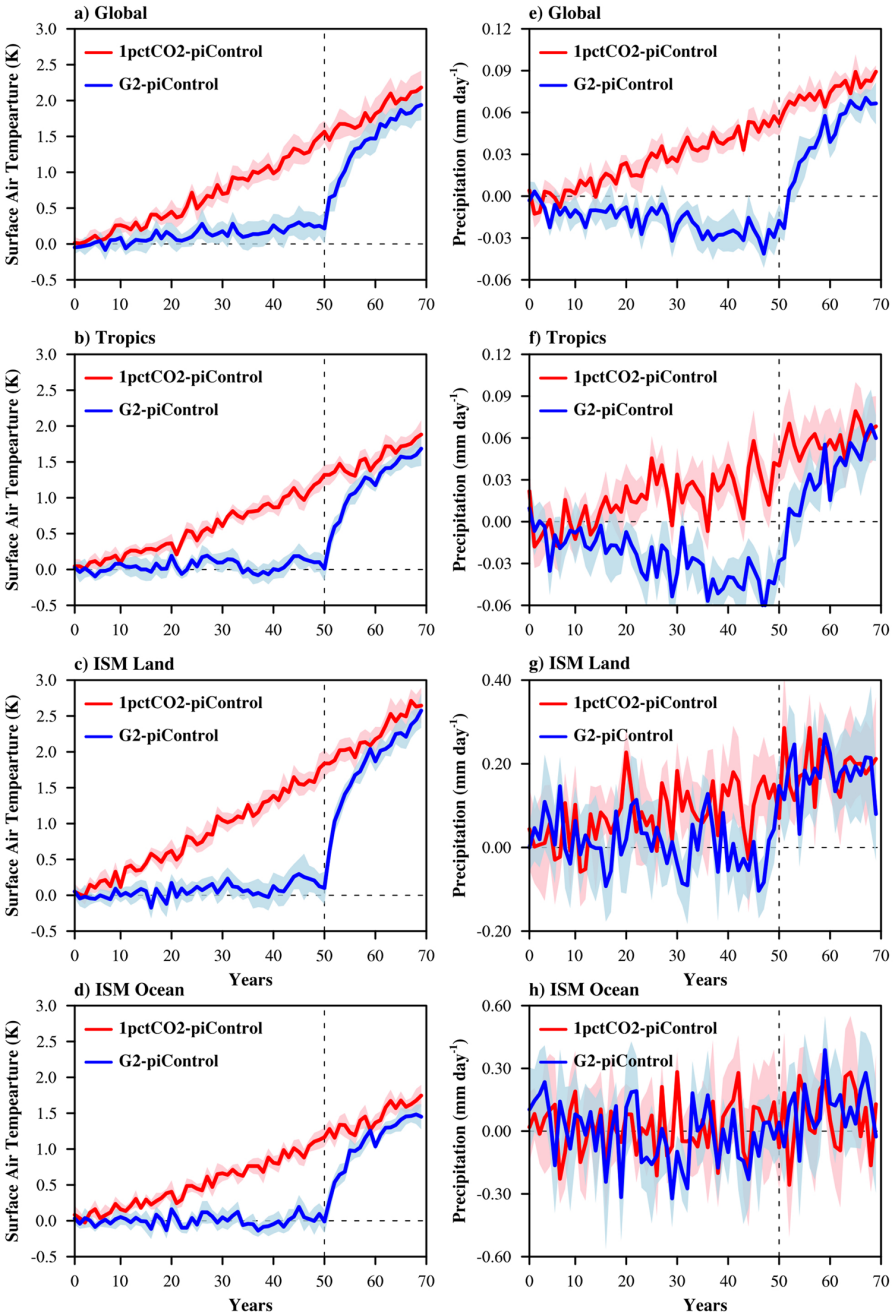
Changes in surface air temperature (°C, left column) and precipitation (mm/day, right column) for 1pctCO2 (red line) and G2 (blue line) simulations relative to the piControl condition for the globe (top row, JJA mean), tropics (middle row, JJA mean), and Indian monsoon region (bottom row, JJAS mean). Lines show the multi-model mean and the shadings show the intermodel standard deviation. The dashed vertical line shows the time of termination of geoengineering in the G2 simulations.

The major portion of the present study evaluates, changes in the mean state of monsoon season temperature, precipitation, moisture and winds for global warming (1pctCO2) and solar geo-engineering (G2) scenarios with respect to (w.r.t.) piControl. Figure 1 compares the change in monsoon season spatial averages of temperature and precipitation over the globe, tropics (30N–30S) and ISM region (15S–45N, 40E–120E) under the 1pctCO2 and G2 scenarios. The global and tropical region monsoon season spans June through August, whereas the monsoon season in the ISM region spans June through September. The shading depicts one standard deviation of inter-model spread. As popularly noted in past global warming studies^3^, this analysis also shows a steady increase in global mean temperature (red line) with CO_2_ concentration increase, reaching ~ 1.9 K (model mean) after 70 years. Mean warming over tropical regions is lower than global mean warming, consistent with polar amplification^20^. Over the ISM region, the warming over the 70-year simulation of 1pctCO2 is similar to that of the global mean warming. Under G2 (blue lines Fig. 1), global warming is compensated well by G2 with residual warming of 0.3 K for the global average, the tropics and the ISM land region. However, over ISM ocean there is no systematic change. The precipitation decreases globally and over the tropics up to0.9 and 1.1%, respectively. With sudden termination of geoengineering (vertical dotted line), global mean temperature is projected to increase rapidly, approaching the outcome of seven decades of continued warming, but in just two decades; these results are aligned with the findings of one of the previous study^9^. The rate of temperature increase over the period after termination is approximately 1.5 °C in the first decade and 0.5 °C in the second. Similar to the temperature response, after abrupt termination of geoengineering, mean precipitation over the globe and tropics show a rapid rate of increase. However, the rates for domain average precipitation change over the ISM land and oceanic region in the geoengineered period and the period after termination are difficult to differentiate due to the large inter-annual variability.

### Changes in surface air temperature

**Figure 2:**
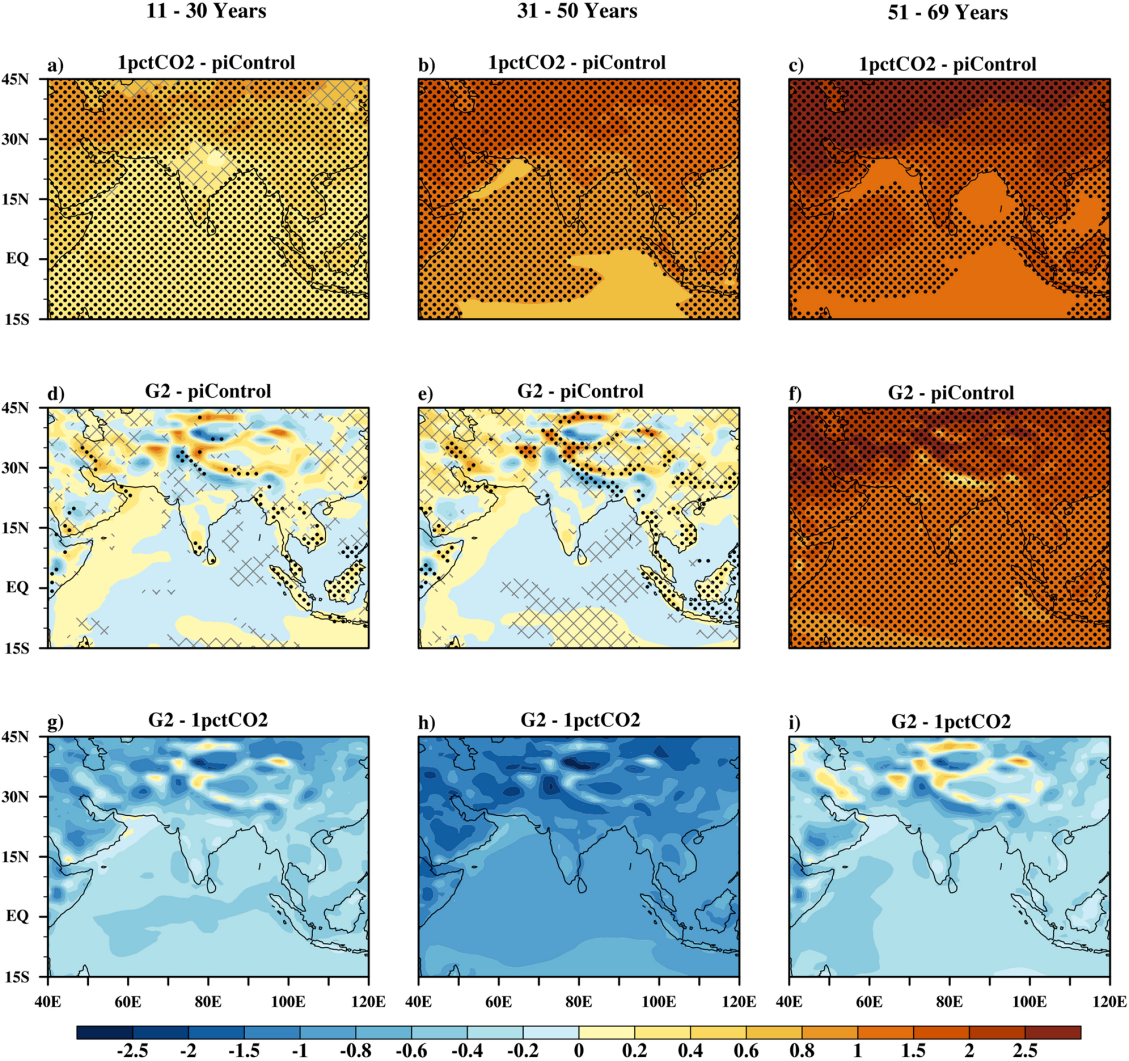
Multi model mean surface air temperature change (^0^C) in the 1pctCO2 simulations (top row), G2 simulations (middle row) relative to piControl simulations for the Indian monsoon season (Jun-Sep) for three consecutive 20 year time periods. Bottom row shows differences between G2 and 1pctCO2. Hatching are the regions that have at least 70% model agreement. Stippling shows changes that are having at least 70% model agreement and also significant at the 95% confidence level.

Figure 2 shows the spatial distribution of multi-model mean surface air temperature anomalies over the ISM region for the 1pctCO2 and G2 simulations with respect to piControl. In the bottom panel, 1pctCO2 and G2 are directly compared. Stippling show changes significant at the 95% confidence level, as calculated by Student’s t-test using the inter-annual variability and where, at least 70% models agrees on the sign of the change. Over years 11–30, the 1pctCO2 scenario (Fig. 2a) projects a statistically significant warming over ISM oceanic regions. However, over the land region, at least 70% models agreed on the sign of change that is positive but values are insignificant over central India and the majority of the sub-tropical land. With further increase in CO_2_ at the rate of 1% per annum for 31–50 years period (Fig. 2b), ocean and land regions show different degrees of warming, resulting in enhancement of the temperature gradient between the land and ocean. During years 51–69 under 1pctCO2 (Fig. 2c), warming gets intensified over both the land and the ocean. Warming is more intense over the Arabian Peninsula and the subtropics of the ISM region as compared to other parts of the Indian sub-continent. Similarly, the northwest equatorial Indian Ocean is projected to be warmer than the rest of the oceanic region. Correspondingly, the land–ocean temperature gradient gets intensified during this period.

Under G2, during the years 11–30 (Fig. 2d,g), surface air temperature remains within 0.25 °C of pre-industrial levels over the ISM region. However, the majority of the Himalayan range is projected to be significantly cooler than other parts of Indian sub-continent. With further sustention of geo-engineering (31–50 years period, Fig. 2e,h), more regions are projected to be significantly cooler than in the corresponding 1pctCO2 scenario. A greater degree of cooling is projected over the Himalayan range than other parts of the ISM region, which may reduce Himalayan glacier melt^21^. Compared to the Indian land, projections over the subtropics are mixed and less conclusive. Similar to the Indian land, the areal extent of significantly cooler regions over ocean, increases with prolonged sustention of geoengineering. These different level of cooling over different regions may be attributed to the presence of inhomogeneous topographic surfaces. With abrupt termination of geoengineering (Fig. 2f,i), temperature over the ISM region rises at a higher rate than in the 1pctCO2 scenario. The warming over the subtropics reaches the 70-year value of the 1pctCO2 simulation within just 20 years. Over the Indian land and oceanic regions, warming after abrupt termination of geoengineering is 0.5—1 °C less than in the sub-tropical regions. This fast rate of warming of subtropical regions (consisting only of land regions) compared to tropical region (partly land and partly ocean) may be attributed to the faster rate of warming of land regions than oceanic regions. However, over tropical Indian land the fast rate of increase in temperature is partly compensated by the cooling effect caused due to increase in precipitation, so the rate of increase of temperature over Indian land is relatively slower than subtropical land.

### Changes in atmospheric circulation

**Figure 3:**
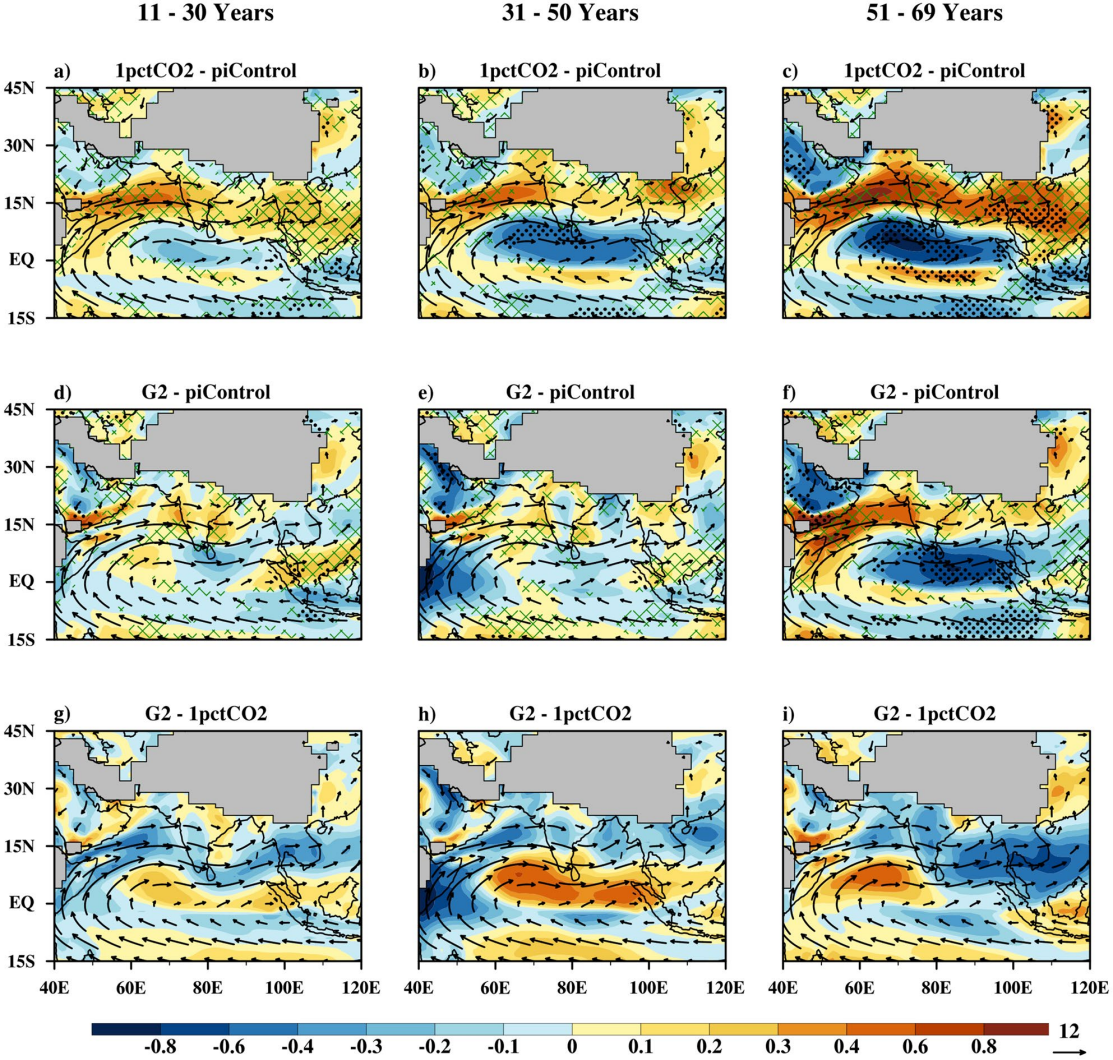
As in Fig. 2 but for wind speed (m/s) at 850 hPa. Vectors show piControl winds at same level.

**Figure 4:**
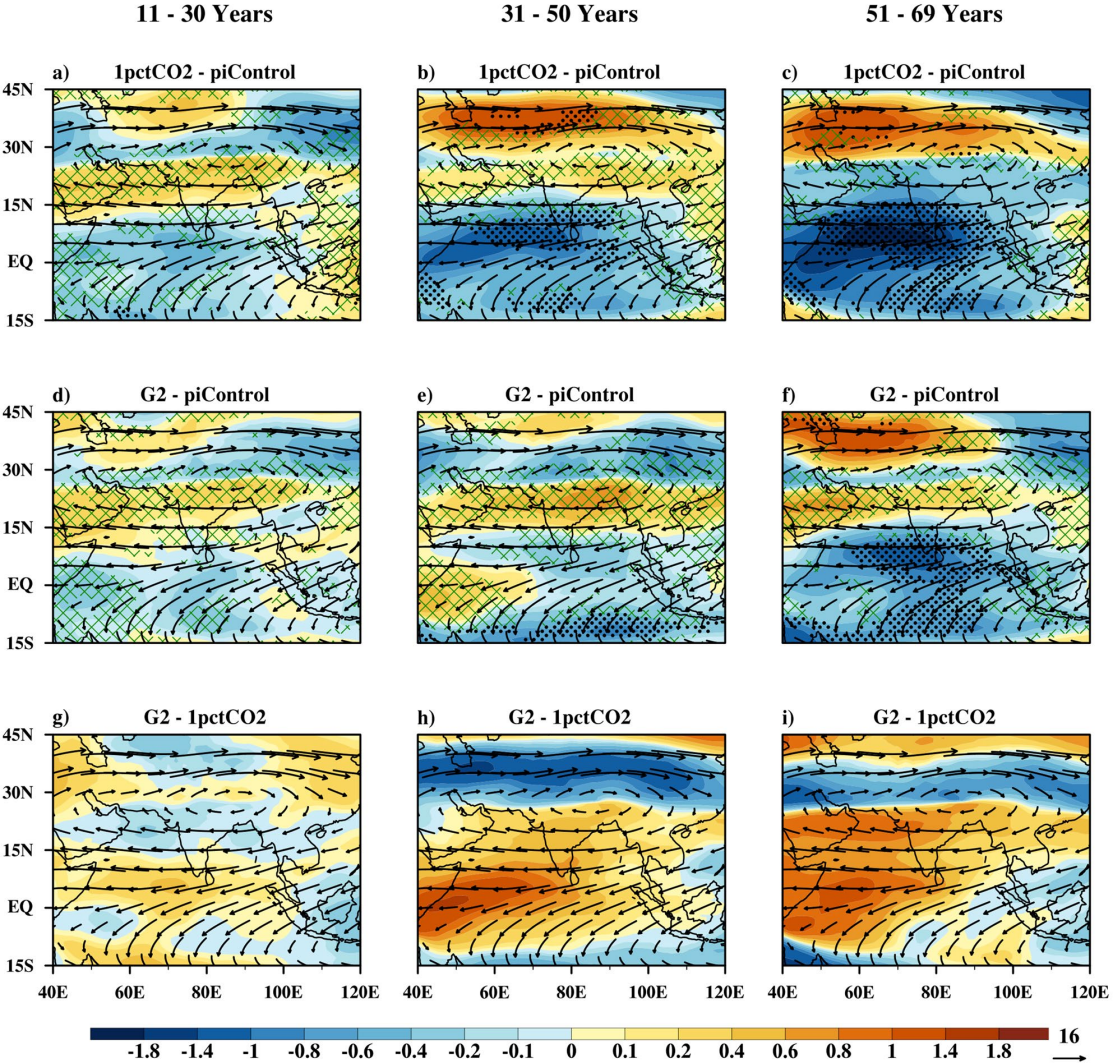
As in Fig. 2 but for wind speed (m/s) at 200 hPa. Vectors show piControl winds at same level.

Figures 3 and 4 demonstrate the response of monsoon wind circulation to global warming, geoengineering and its termination. The low level (850 hPa) wind features that are conducive to a normal monsoon season include setting up of the monsoon trough over the Gangetic plain, evolution of cross-equatorial flow, and setting up of the Somali Jet^22^, over Arabian Sea traversing to Bay of Bengal. Apart from this, central and southern India is dominated by moist south-westerlies. Wind features at the upper level (200 hPa) during the summer monsoon include a northward shift of the Subtropical Westerly Jet (STWJ^23^) and setting up of the Tropical Easterly Jet (TEJ) over the Bay of Bengal and southern peninsula^24^.

#### Low level circulation

In years 11–30 of the 1pctCO_2_ scenario (Fig. 3a), an increase in low-level wind speed is projected over the majority of Indian land except near the eastern Gangetic plain. Over the same period, a decrease in the speed of winds is projected near the equator, Indo China, and parts of the Arabian Peninsula. However, the respective increase and decrease in low level wind speed is not statistically significant for this period. In the subsequent 20-year period (years 31–50, Fig. 3b), the cross-equatorial flow and Somali jet is projected to strengthen w.r.t piControl. For years 51–69 (Fig. 3c), an increase in wind speed over Indian land is projected by at least 70% of the models. The Somali jet speed over the Arabian Sea is also projected to increase but, insignificantly with an increase in global warming, as projected by at least 70% of the models.

Under scenario G2, for 11–30 years period, the geoengineering shows a good amount of compensating effect to global warming in wind speed (Fig. 3d). A significant decrease in wind speed over the east equatorial region is projected with further sustainment of geoengineering (31–50 years period, Fig. 3e). The Somali jet speed increases considerably during this period w.r.t piControl. With abrupt termination of geoengineering (Fig. 3f,i) in the Somali Jet and associated regions, wind conditions are slightly under compensating compared to the global warming scenario. However, a significant decrease in low-level wind speed over the Arabian Peninsula is simulated, after abrupt termination of geoengineering.

#### Upper level circulation

The upper level winds under 1pctCO_2_ in the 11–30 years period (Fig. 4a) show weak increase in the speed as compared to the piControl, over the southern Peninsula and central India by at least 70% of the models. Over the years 31–50 (Fig. 4b), a significant increase in wind speed of STWJ is projected. In contrast, the TEJ is projected to weaken significantly. Further enhancement in greenhouse gas forcing (51–69 years period, Fig. 4c) results in a strengthening of the STWJ, but the TEJ and winds over the equatorial Indian Ocean weaken significantly.

Under G2, in the 11–30 years period (Fig. 4d,g), no significant changes in upper level wind speed is projected. With prolonged sustention of solar geoengineering (31–50 years period, Fig. 4e,h), wind speeds over southern India, central India, northeast India, Myanmar, the head of the Bay of Bengal, the Arabian Sea, and southwest equatorial Indian Ocean are projected to increase by at least 70% of the models. The STWJ is projected to strengthen under prolonged solar geoengineering. The TEJ over the southern India is projected to weaken over this period. In general, under solar dimming, the STWJ and upper level monsoon circulation are projected to intensify except TEJ. Abrupt termination of geoengineering (Fig. 4f,i) shows characteristics similar to the 1pctCO_2_ simulation: weakening of the TEJ and strengthening of the STWJ.

### Low level moisture convergence

**Figure 5:**
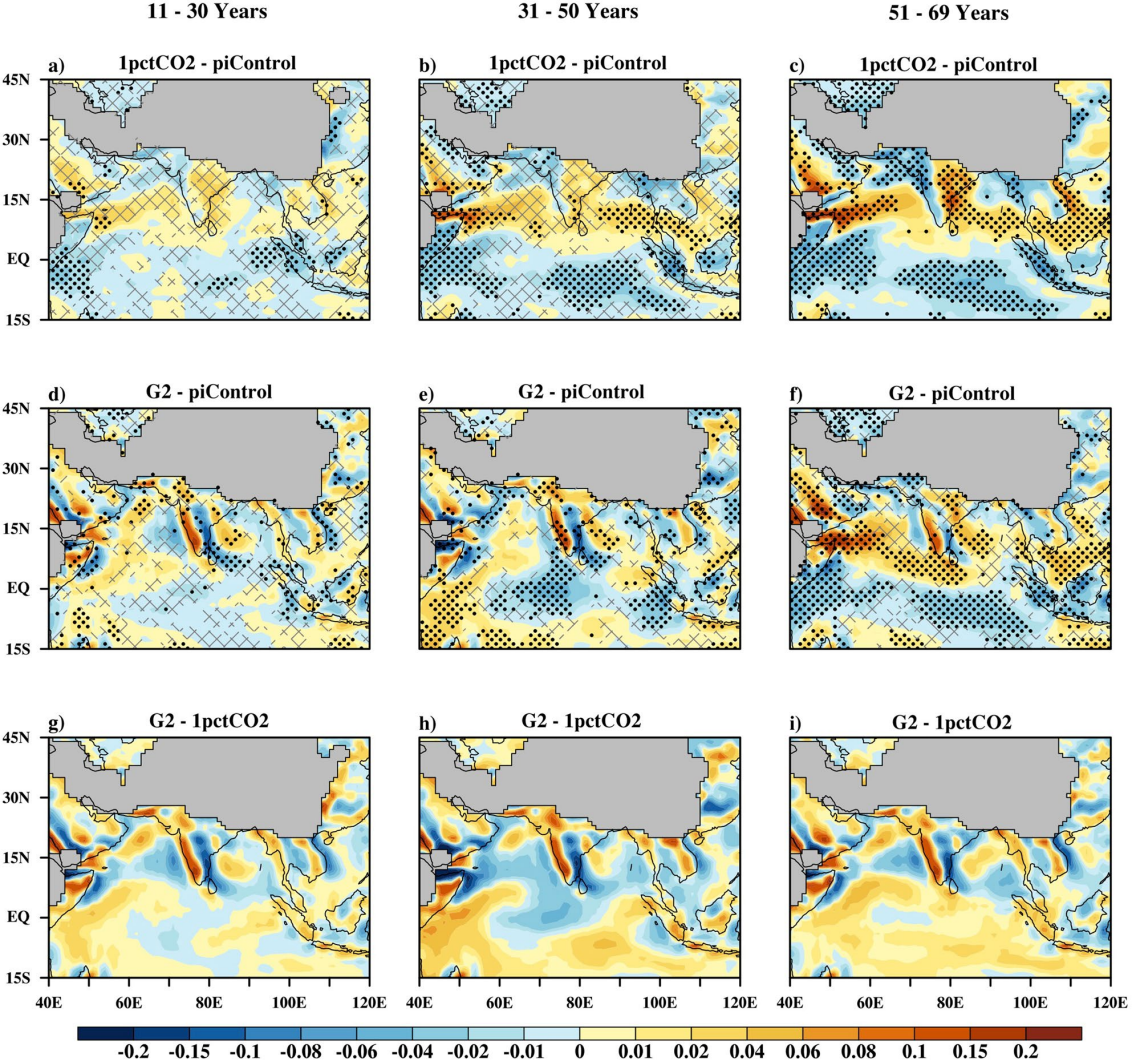
As in Fig. 2 but for moisture convergence (X 10^4^) at 850 hPa (g/Kg s).

Top and middle panels of Fig. 5 show projected low level (850 hPa) moisture convergence anomaly of 1pctCO2 and G2 w.r.t piControl. The bottom panels of the figure show differences between the G2 and 1pctCO2. In the 11–30 years period under 1pctCO2, at least 70% of the models agree on the sign of the change in convergence anomaly. Moisture convergence increases over the Arabian Peninsula, central, south-east India and eastern Arabian Sea. However, over equatorial Indian Ocean, including maritime continent, moisture convergence decreases significantly. In the subsequent years (31–50 years, Fig. 5b), regions of statistically significant anomalies increases. Strong moisture convergence is projected over the eastern Arabian Sea, southern peninsula, Bay of Bengal and parts of the maritime continent. In the period, 51–69 years, monotonic increase or decrease in moisture convergence is projected. Eastern Arabian peninsula, western Arabian Sea, south east India, southern Bay of Bangal and parts of north maritime continent show significant positive anomaly. In contrast, Western Ghats, western India, and south east equatorial Indian Ocean are projected to have decrease in moisture convergence.

Under G2, in the first two 20 years period of geoengineering (Fig. 5d,e), the residual heating in Somali coast led to increase in Somali jet speed and hence increases evaporation over the Arabian Sea. This increase in moisture leads to increase in moisture convergence near Western Ghats. Once the convection is triggered, it leads to a locally enhanced tropospheric temperature gradient leading to a stronger Somali Jet. Since the moisture carried by the Somali Jet is not able to reach the Indian land due to preferential precipitation over the windward side of Western Ghats, a dry bias is noted over majority of the Indian land. In the year 51–69 (Fig. 5f), strong moisture convergence is observed over majority of Somali Jet region, western Ghats and Bay of Bengal. Thus, we have observed that lateral displacement of local high and low Somali Jet speed affects the positioning of moisture convergence.

### Precipitation changes

**Figure 6:**
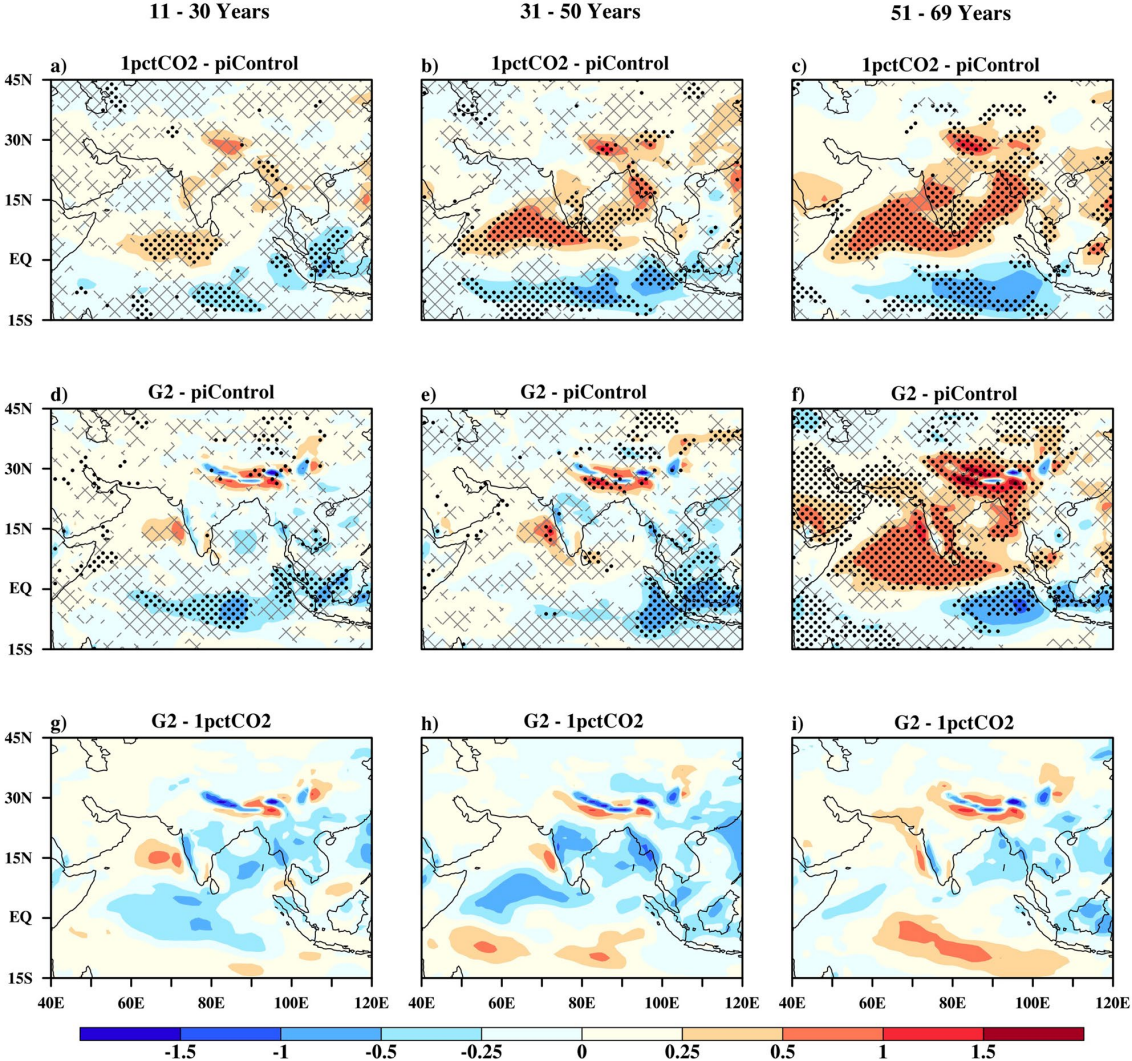
As in Fig. 2 but for precipitation (mm/day).

JJAS mean precipitation changes for 1pctCO2 and G2 w.r.t piControl are shown in Fig. 6. The stippling and hatch criterion is the same as for surface air temperature. Over the 11–30 years period under the 1pctCO_2_ scenario (Fig. 6a), a statistically significant increase in precipitation is projected for some parts of Myanmar and the equatorial Indian Ocean. Whereas, a marginal, insignificant increase in precipitation is projected over the majority of the Indian land however, at least 70% of the models agree in the sign of change that is positive. Over the subsequent 20 years period (years 31–50, Fig. 6b), a statistically significant increase in precipitation is projected over parts of the northeast Himalayan range and the mountains of Myanmar. The region near the equatorial Indian Ocean is projected to have an increase in precipitation, coincident with weakening of the TEJ. Over the south equatorial Indian Ocean and the maritime continent, precipitation is projected to decrease within this second 20 years period. For the 51–69 years period (Fig. 6c), precipitation is projected to increase over some parts of the Himalayan range, interiors of the southern peninsula, Myanmar, and parts of the Arabian Peninsula. The Bay of Bengal and north Indian Ocean are also projected to have significant increases in precipitation under the 1pctCO2 scenario. However, the regions to the south of equatorial Indian Ocean and the maritime continent are projected to have a significant decrease in precipitation in both 1pctCO2 and G2 scenarios.

Under the G2 scenario, the majority of Indian land shows an insignificant decline in precipitation relative to piControl (Fig. 6d-e). However, precipitation is projected to increase over the east Arabian Sea. Exceptions include near the Himalayas, where results are more regionally variable. With abrupt termination of geoengineering (see Fig. 6f), the rebound of warming leads to a rapid rise in precipitation over the majority of the ISM region. Parts of the Gangetic plain, the southern Peninsula, the Arabian Peninsula, and Bangladesh are projected to have statistically significant increases in precipitation, as are the Bay of Bengal and the north Equatorial Indian Ocean. Similar to the 1pctCO2 scenario (Fig. 6c), the south equatorial Indian Ocean and maritime continent are projected to receive less rainfall as compared to preindustrial levels after the termination of geoengineering.

## Mechanisms of change and conclusions

Response on the mean conditions of the Indian summer monsoon to global warming (1% per annum rising rate of CO_2_), solar geoengineering (gradual reduction of the solar constant to balance the changing radiative forcing due to the rising rate of CO_2_), and its abrupt termination are analyzed in this study. Because the Asian monsoon-system involves complex feedbacks^25^, finding cause-and-effect relationships is not simple. Also, over the ISM region, domain average multi model mean inter annual variability (Fig. 1) tends to be potentially obscuring the mechanisms. Nevertheless, by examining spatial changes in temperature, precipitation, wind fields, and moisture convergence, we are able to ascertain some conclusions about how the ISM may change under idealized scenarios.

Under global warming, surface air temperature over land increases more than over ocean, strengthening the well-documented summer season land ocean temperature gradient^26, 27^, thus strengthening the low-level (near 850 hPa) monsoon circulation. This strengthening of low level monsoon circulation (Somali jet) brings in more moisture and enhanced evaporation over land due to rise in temperature, resulting in enhancement of precipitation near the west coast of India, east Arabian Sea and peninsular India. At upper levels, the TEJ is projected to progressively weaken under global warming, likely resulting in the reduction of vertical wind shear. The reduced wind shear conditions would enable more depressions to become cyclones in the Bay of Bengal^28^, and correspondingly increase precipitation and associated wide spread flood conditions in the region near the east coast of India. This weakening of the TEJ can be associated with the reduction in the magnitude of the meridional temperature gradient (Supplementary Fig. 1) at upper levels due to the warming of the upper troposphere over the equatorial Indian Ocean caused by latent heat release from enhanced convection^29, 30^. In contrast, over Indian longitudes (60 to 100°E), the STWJ strengthens and shifts southward under the global warming scenario but its effect (decrease in precipitation) seems to be over shadowed by the above-discussed effects of the TEJ and the Somali Jet. Thus, under global warming, increase in the strength of the low-level monsoon circulation and higher amounts of moisture in the atmosphere^31^ both result in enhancement of precipitation over the ISM region.

On the other hand, solar geoengineering under experiment G2, compensates the global average surface warming and may cause a slight cooling over the majority of the ISM region. At upper levels, the TEJ and STWJ are projected to strengthen. At lower levels, Somali Jet is projected to strengthen but spatially irregularly. This strengthening of the Somali jet is likely to be due to residual warming near the Somali coast. The precipitation is projected to increase near the windward side of the Western Ghats and decrease over majority of the Indian land. The preferential precipitation over the windward side of the Western Ghats could likely be due to increased moisture convergence in the Somali Jet exit region. The initial triggering of precipitation due to moisture convergence over the eastern Arabian Sea and the west coast of India, may then sustain the Somali Jet by feedback effects. Since the moisture carried by the Somali Jet is not able to reach the Indian land due to preferential precipitation over the windward side of Western Ghats, and reduction of sunlight at the surface leads to less evapo-transpiration leads to less water vapour and thus a dry bias is noted over Indian land^17, 32^. At upper levels, the projected residual cooling of the tropics and residual heating of the subtropics (Supplementary Fig. 1) results in strengthening of the meridional temperature gradient, thus increasing TEJ speed. Thus, it is noted that wind (monsoon) circulation at upper and lower levels gets strengthened in scenario G2. However, precipitation over the majority of the ISM region is reduced. This result agrees with some of the previous^17, 32, 33^ findings that ISM precipitation will decrease under solar reduction scenario. Abrupt termination of the geoengineering results in a sudden increase in temperature throughout the globe. Over the ISM region, termination of geoengineering is found to induce conditions similar to a prolonged global warming condition with a strengthening of the Somali Jet and weakening of TEJ, resulting in an increase of precipitation over the ISM region.

While the analysis presented here utilizes a multi model mean approach, the presence of very limited statistically significant regions under the geoengineered scenario highlights the need for regional studies in the context of geoengineering with large ensembles of targeted numerical modeling experiments. Further, with sudden termination of G2 leads to large increases in temperature and increases in precipitation indicate a potential risk of drought and wide spread floods respectively. Thus, many more downstream climate impact studies are needed for a more holistic assessment at the socioeconomic level.

## Data and methodology

The data used in this study is from Coupled Model Intercomparion Project (CMIP) and Geoengineering Model Intercomparison Project (GeoMIP). All the data was downloaded from Earth System Grid Federation site (https://esgf-index1.ceda.ac.uk/projects/esgf-ceda/). Kindly refer to Table 1 for the detail of the models and variables used.

In the spatial analysis, we have taken averages for the entire pre- industrial period (and for each future period) for each model and then have 8 ‘years’ for each simulation. Two statistical tests are performed in Figs. 2, 3, 4, 5, 6 to find the robustness of multi model mean changes. In the first test, model agreement is computed from the 8 ‘years’ of simulation as 70% model agreement in change of sign of different experiments (1pctCO2 and G2) w.r.t piControl. Second test is student t-test on multi model mean changes, significant at 95% or more in confidence level using inter annual variability. Data of all models are re-gridded to 1° × 1° using bilinear interpolation for computational purposes. Figures are created using the NCAR/NCL33 software version 6.4.0, https://www.ncl.ncar.edu.

## Supplementary Information

Supplementary Information
