# Supplementary material for: Response of the Indian summer monsoon to global warming, solar geoengineering and its termination
Source: Sci Rep. 2021 May 7;11:9791. doi: 10.1038/s41598-021-89249-6 (PMC8105343; doi:10.1038/s41598-021-89249-6)
Supplement: Supplementary file 1 — Supplementary Information [file 41598_2021_89249_MOESM1_ESM.pdf]

Response of the Indian Summer Monsoon to Global Warming, Solar Geoengineering and its Termination

Mansi Bhowmick[1,*], Saroj Kanta Mishra[1], Ben Kravitz[2,3], Sandeep Sahany[1], Popat Salunke[1]

[1]Centre for Atmospheric Sciences, IIT Delhi, India
[2]Department of Earth and Atmospheric Sciences, Indiana University, Bloomington, IN, USA
[3]Atmospheric Sciences and Global Change Division, Pacific Northwest National Laboratory, Richland, WA, USA

Submitted to Scientific Reports

***Corresponding Address**

Mansi Bhowmick
Centre for Atmospheric Sciences
Indian Institute of Technology Delhi (IIT Delhi)
New Delhi - 110016
INDIA
E-mail: mansibhowmick@gmail.com
Ph: +91-11-26596053

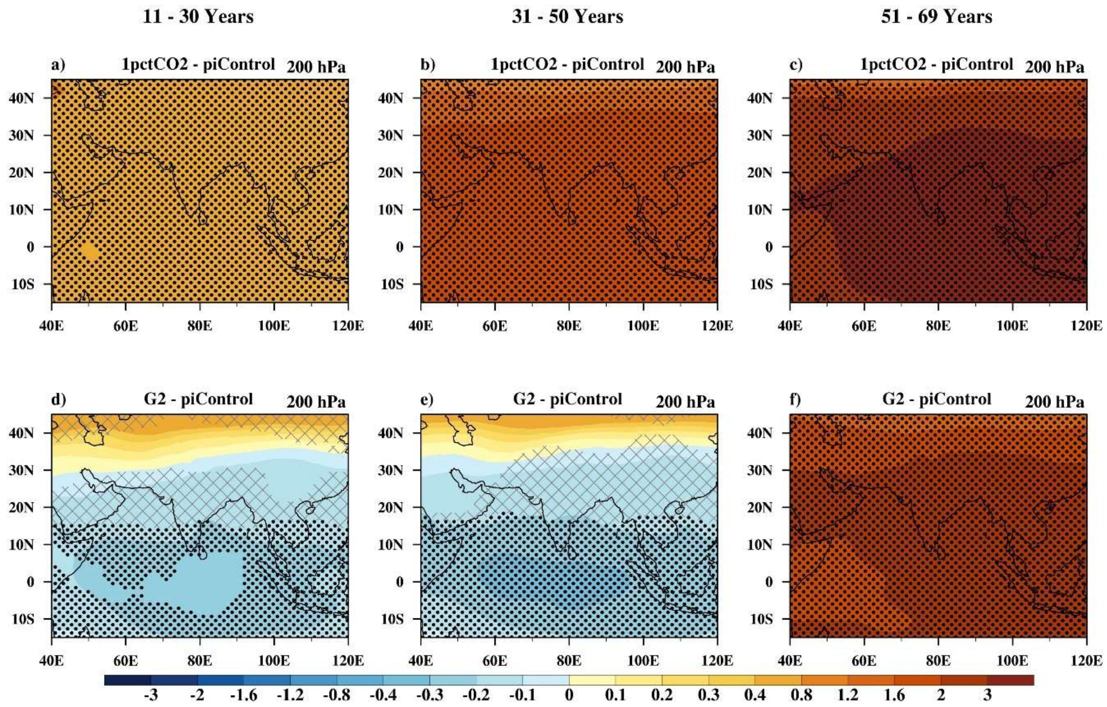

Supplementary Figure 1: Multi model mean 200hPa temperature changes (^{0}C) in the 1pctCO2 simulations (top row) and G2 simulations (bottom row) relative to the piControl simulations for the Indian monsoon season (Jun-Sep) for three consecutive 20 year time periods. Hatching are the regions that have at least 70% model agreement. Stippling shows changes that are having at least 70% model agreement and significant at the 95% confidence level.
